# No-Reference Video Quality Assessment Using the Temporal Statistics of Global and Local Image Features

**DOI:** 10.3390/s22249696

**Published:** 2022-12-10

**Authors:** Domonkos Varga

**Affiliations:** Ronin Institute, Montclair, NJ 07043, USA; domonkos.varga@ronininstitute.org

**Keywords:** no-reference video quality assessment, quality-aware features, multi-feature fusion

## Abstract

During acquisition, storage, and transmission, the quality of digital videos degrades significantly. Low-quality videos lead to the failure of many computer vision applications, such as object tracking or detection, intelligent surveillance, etc. Over the years, many different features have been developed to resolve the problem of no-reference video quality assessment (NR-VQA). In this paper, we propose a novel NR-VQA algorithm that integrates the fusion of temporal statistics of local and global image features with an ensemble learning framework in a single architecture. Namely, the temporal statistics of global features reflect all parts of the video frames, while the temporal statistics of local features reflect the details. Specifically, we apply a broad spectrum of statistics of local and global features to characterize the variety of possible video distortions. In order to study the effectiveness of the method introduced in this paper, we conducted experiments on two large benchmark databases, i.e., KoNViD-1k and LIVE VQC, which contain authentic distortions, and we compared it to 14 other well-known NR-VQA algorithms. The experimental results show that the proposed method is able to achieve greatly improved results on the considered benchmark datasets. Namely, the proposed method exhibits significant progress in performance over other recent NR-VQA approaches.

## 1. Introduction

The recent rise in video-driven data consumption has presented manufacturers and telecommunications service providers with the problem of providing improved video services [1]. Further, it has also created a compelling necessity to monitor and regulate video quality [2]. As a consequence, video quality assessment (VQA) has received more and more attention from both academia [3] and industry [4]. In numerous video processing activities, including video capture, compression, and transport, VQA—which seeks to anticipate the perceived quality of a video—is still a challenging task. Similarly to image quality assessment (IQA), VQA is also divided into two groups, i.e., subjective and objective, in the literature [5]. Subjective VQA involves laboratory and crowdsourcing experiments [6] for collecting quality ratings from human observers by presenting them with various video sequences. Further, objective VQA deals with mathematical and computational models that are able to predict digital videos’ perceptual quality consistently with human quality perception. Although subjective VQA is more reliable than objective VQA, since it collects quality ratings directly from humans, at the same time, it is expensive and time-consuming [7]. This is why it cannot be applied in real-time systems, and objective VQA is a hot research topic. Traditionally, objective VQA methods are further divided in the literature depending on the availability of the reference pristine (distortion-free) videos [8]. Specifically, no-reference (NR) VQA methods have no access to the reference methods, while full-reference (FR) VQA methods have complete access to them. Reduced-reference (RR) VQA methods have partial information about the reference videos. In practice, NR-VQA is highly demanded, since reference videos are unavailable in many real-world applications [9].

Researchers of visual physiology have demonstrated that the human visual system (HVS) tends to produce an unconscious global impression about a scene [10]. Next, the HVS focuses on the local details step by step [11,12,13]. The main contributions of this study are as follows. Based on the previous point, we extract the temporal statistics of both local and global image features for NR-VQA. Namely, the temporal statistics of global features reflect all parts of the video frames, while the temporal statistics of local features reflect the details. Inspired by our previous work [14], we adapt the statistics of local feature descriptors extracted from filtered images for NR-VQA to compile video-level local feature vectors. Namely, several HVS-inspired filters, i.e., Bilaplacian, high-boost, and derivative filters, were introduced to enhance the statistical regularities of an image that influence human quality perception. Specifically, these HVS-inspired filters were first applied over the color channels of a video frame. Next, the statistics of FAST (features from accelerated segment test) [15] feature descriptors were used to compile frame-level features. Video-level features were obtained through the temporal pooling of frame-level features. Further, we propose an ensemble learning framework to integrate the predicted quality scores of several machine learning techniques for efficient quality estimation. Due to the previously mentioned innovations, our experimental results demonstrate that the performance of the proposed method surpasses that of other recently published NR-VQA methods on two large VQA benchmark databases, i.e., KoNViD-1k [16] and LIVE VQC [17], which contain authentically distorted video sequences.

The following is the paper’s flow. Section 2 reviews related and previous work. The proposed method is discussed in Section 3. Subsequently, Section 4 describes our experimental results and a comparison with the state of the art. Our conclusion is in Section 5.

## 2. Literature Review

Recent NR-VQA techniques can be classified into two broad categories: (i) those that only take into account spatial image-level characteristics and (ii) those that also take into account the temporal information between a video’s frames [18]. Further, the majority of many modern NR-VQA methods apply some kind of machine or deep learning technique.

Image-based NR-VQA techniques borrow many ideas from NR-IQA and analyze the natural scene statistics (NSS) for quality prediction. The assumption behind NSS is that natural scenes follow certain statistical regularities that are distorted in the presence of image noise [19]. In the case of video data, many NSS-based algorithms independently measure frame-by-frame deviations from the “natural” statistics [20,21,22]. In [23], five simple perceptual features (blurriness, contrast, colorfulness, spatial information, temporal information) were determined frame by frame and temporally pooled to construct a video-level feature vector, which was mapped onto perceptual quality scores with a trained support vector regressor (SVR) [24]. Other approaches also took temporal information into consideration in addition to temporal pooling [25]. For instance, the image-based metric was developed further by V-BLIINDS [26], which incorporated time–frequency and temporal motion information as well. In contrast, Yan et al. [27] extracted features, i.e., moments of feature maps, gradient magnitudes’ joint distributions, filtering responses of Laplacians of a Gaussian, and motion energy, from multi-directional spatiotemporal slices and mapped them onto quality scores with either a shallow neural network or an SVR. Similarly, Lemesle et al. [28] combined frame-level and video-level features for NR-VQA. After testing a wide combination of features, the authors concluded that the histogram of oriented gradients [29], edge information, fast Fourier transform [30], blur, contrast, freeze, and temporal-information-based features were the most informative ones for predicting video quality without a reference. Instead of perceptual features, Wang and Li [31] devised a statistical model for the speed perception of the human visual system, which was utilized for the estimation of motion information and perceptual uncertainty. Contrarily, Hosu et al. [16] introduced several video-level perceptual features and mapped them onto perceptual quality scores with the help of an SVR [24].

Deep learning has recently been utilized for NR-VQA. One of the first methods utilizing deep learning was SACONVA [32], which extracted feature vectors from video data via a 3D shearlet transform [33]. Next, these features were mapped onto quality scores using logistic regression and a convolutional neural network (CNN). In contrast, Wang et al. [34] combined deep spatial and temporal features for perceptual quality prediction. Specifically, spatial features were obtained through the pooling of a CNN’s activations. Further, the standard deviations of motion vectors were considered as temporal features. Next, two predictions were obtained from these two sets of features, and they were combined by using a Bayes classifier for video quality prediction. Agarla [35] proposed an approach in which the image quality attributes, i.e., sharpness, graininess, lightness, and color saturation, of video frames were estimated first by using the deep features of a CNN. Based on these attributes, frame-level quality scores were estimated. Finally, a recurrent neural network was trained for video quality estimation by using the previously predicted frame-level scores as training data. The two-level video quality model (TVLQM) proposed by Korhonen [36] first computed low-complexity features from the entire video sequence before the extraction of high-complexity features. Further, the author fused traditional hand-crafted temporal features with deep features extracted from a CNN, which was trained to predict digital images’ perceptual quality. Similarly, Agarla et al. [37] extracted frame-level quality-aware features by using pretrained CNNs, but they introduced a temporal modeling block containing a recurrent neural network (RNN) [38] and a temporal hysteresis pooling for quality prediction. Chen et al. [39] also applied RNNs for NR-VQA. To be more specific, this method consisted of two steps: (i) learning of quality degradation and (ii) modeling of motion effects. Similarly to the previously mentioned algorithms, the authors used CNNs for deep feature extraction. Further, a hierarchical temporal model that included an RNN was introduced for temporal down-sampling and gathering of motion information. Li et al. [40] took a similar approach, but they used a gated recurrent unit (GRU) [41] that was trained on the deep features extracted from a ResNet [42] network for perceptual quality estimation. This method was further improved by Zhang and Wang [43] provided texture features aside from deep features. In contrast, Chen et al. [39] extracted motion information from different temporal frequencies and trained a hierarchical recurrent network for video quality estimation. Contrary to the previously mentioned approaches, Li et al. [44] experimented with the idea of a mixed-dataset training strategy to improve the performance of NR-VQA by increasing the size of the training database and to boost the generalization capability of the implemented model. Further, this model was trained by two different loss functions, i.e., monotonicity- and linearity-induced loss. In [45], the authors first implemented a visual attention module that obtained frame-level perceptual quality scores. Next, video quality predictions were obtained with the help of a structure imitating human visual and memory attention.

## 3. Proposed Method

**Figure 1:**
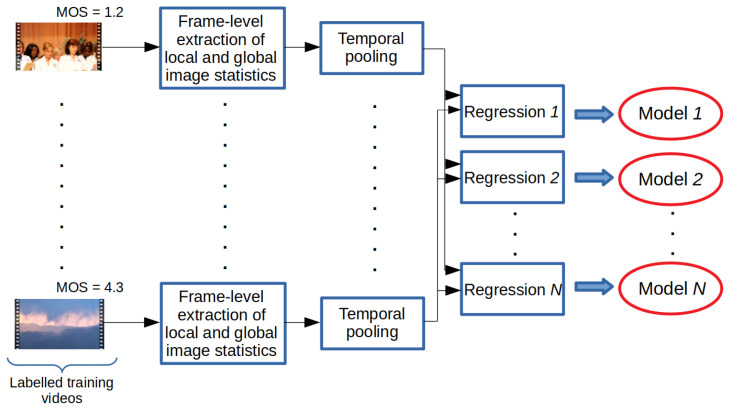
Training process of the proposed method. Video-level feature vectors are obtained from labeled training videos through the temporal pooling of local and global image statistics. Next, several regressors are trained.

**Figure 2:**
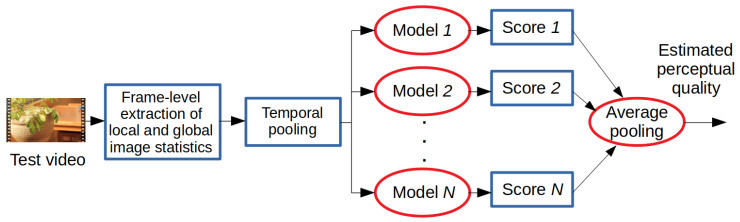
Testing of the proposed method. Video-level feature vectors are extracted from a test video through the temporal pooling of local and global image statistics. The scores of the trained regressors are fused together via average pooling to get an estimation of the perceptual quality.

The training and testing processes of the proposed method are summarized in Figure 1 and Figure 2. In the training stage, the statistics of local and global image features were extracted from each frame of a video sequence found in the training database. Subsequently, these image statistics were temporally pooled together to compile a quality-aware feature vector that characterized a given video. Based on the extracted video-level feature vectors, several different machine learning models, i.e., a generalized additive model (GAM) [46], an LSBoost algorithm [47], a Gaussian process regressor (GPR) [48] with rational quadratic kernel function, a neural network (NN) with one hidden layer containing 10 neurons [49], an SVR with a radial basis function (RBF) [24], a binary decision tree (BDT) [50], and an extra tree (ET) [51], were trained for perceptual quality estimation. In the testing stage, these trained models were used to generate quality scores for a previously unseen video. The final quality score was obtained by taking the arithmetic mean of the models’ scores. In Section 3.1 and Section 3.2, the processes of the extraction of global and local features are given. Further, in an ablation study (Section 4.2), we provide proof that the proposed ensemble framework results in improved performance compared to the performance of the individual regressors.

### 3.1. Global Features

Many quality-aware features that characterize an image globally have been proposed in the literature in recent decades [52]. Due to their low computational complexities, BRISQUE [21], OG-IQA [53], SSEQ [54], and GM-LOG-BIQA [55] were utilized to compile video-level features through temporal pooling of their statistics. Specifically, BRISQUE [21] extracts features in the spatial domain. First of all, the mean subtracted normalized coefficient of an image is determined. Next, an asymmetric generalized Gaussian distribution (AGGD) is fitted to these coefficients. The parameters of the AGGD were considered quality-aware features. In contrast, OG-IQA [53] uses the variances in gradient magnitude, gradient orientation, and relative gradient magnitude maps as a feature vector. SSEQ [54] utilizes the spatial and spectral (discrete cosine transform coefficients) entropies of an image. GM-LOG-BIQA [55] compiles the joint distribution of the gradient magnitude and Laplacian features for quality-aware feature extraction. To define a global video-level feature vector, the previously mentioned quality-aware features were first determined for each video frame. Next, several well-known statistics, i.e., mean, median, standard deviation, entropy, skewness, and kurtosis, were extracted from a frame-level quality-aware feature. The arithmetic means of these statistics over time were considered as the video-level quality-aware features. As a result, a vector with a length of 24 could be obtained for a single video sequence.

To boost the performance of the applied global features, the following set of perceptual features was also incorporated into our model.

1. Blur: This refers to the parts of an image that are out of focus. With too much blur, edges are no longer distinct. As a consequence, the amount of blur is an important element of human perceptual judgment. Due to its low computational complexity, the metric of Crété-Roffet et al. [56] was chosen in our model for the characterization of the amount of blur in a video frame. A video sequence’s blur was defined as the average of all video frames’ blur.
2. Colorfulness (CF): This is a characteristic of human visual perception that describes whether an image or image area seems to be more or less chromatic [57]. In [58], it was pointed out that humans tend to have a tendency toward more colorful scenes. In our model, we adopted the definition of colorfulness for a video frame proposed by Hasler and Suesstrunk [59]:
  (1) $$CF=\sqrt{\sigma_{rg}^{2}+\sigma_{yb}^{2}}+\frac{3}{10}\sqrt{\mu_{rg}^{2}+\mu_{yb}^{2}},$$
  where rg=R−G and $yb=\frac{1}{2}\left(R+G\right)-B$. Further, *R*, *G*, and *B* denote the red, green, and blue color channels, respectively. The variables of μ and σ stand for the means and standard deviations of the matrices given in the subscripts, respectively. A video sequence’s colorfulness was considered as the average of all video frames’ colorfulness.
3. Vividness was suggested as a color attribute by Berns [60], and it describes the degree of departure of the color from a neutral black color. Berns’ model can be expressed by the following formula:
  (2) $$V_{B}=\sqrt{{\left(L^{*}\right)}^{2}+{\left(a^{*}\right)}^{2}+{\left(b^{*}\right)}^{2}},$$
  where $L^{*}$, $a^{*}$, and $b^{*}$ correspond to the color channels’ values in the CIELAB color space [60,61]. In this study, the vividness of an image was defined by the average of all $V_{B}$ values calculated from CIELAB’s channels. As a quality-aware feature for a video sequence, the average of all video frames’ vividness was taken.
4. The heaviness of a given color is also expressed with the help of the CIELAB space [62,63]:
  (3) $$H=3.8-0.07\cdot L^{*}.$$
  In this study, the heaviness of an image was defined by the average of all *H* values calculated from CIELAB’s channels. As a quality-aware feature for a video sequence, the average of all video frames’ heaviness was taken.
5. Depth is also a color attribute, but it characterizes the degree of departure of a given color from a neutral white color, and in Berns’ model [60], it is formally given as:
  (4) $$D_{B}=\sqrt{{\left(100-L^{*}\right)}^{2}+{\left(a^{*}\right)}^{2}+{\left(b^{*}\right)}^{2}}.$$
  In this study, the depth of an image was defined by the average of all $D_{B}$ values calculated from CIELAB’s channels. As a quality-aware feature for a video sequence, the average of all video frames’ depth was taken.
6. The spatial information (SI) of a video frame is defined with the help of the non-maximum suppression (NMS) [64,65] algorithm. Namely, a video frame is characterized as the number of detected local extrema using three different *T* thresholds (T=1, T=15, and T=30 were considered in this study). More specifically, the filtered video frame in which NMS is carried out is defined as follows:
  (5) $$F\left(x,y,T\right)=\left\{\begin{array}{cc} 1, & \mathrm{if}\;\forall_{\left(x,y\right)}I\left(x,y\right)>I\left(x+i,y+j\right)+T,\\ 1, & \mathrm{else}\;\mathrm{if}\;\forall_{\left(x,y\right)}I\left(x,y\right)<I\left(x+i,y+j\right)-T,\\ 0, & \mathrm{otherwise} \end{array}\right.$$
  where I(x,y) represents the value of pixel intensities at location (x,y). Further, (i,j)∈{(0,1),(0,−1),(1,0),(−1,0)}. In other words, the 3×3 neighborhood around (x,y) is considered. The SI of a video frame was defined as the entropy of the detected extremes’ pixel intensities by using the three different previously mentioned thresholds. As a quality-aware feature for a video sequence, the average of all frames’ SI was utilized.
7. Temporal information was defined by using the difference between two consecutive video frames. Namely, the standard deviations of all difference maps were determined, and their arithmetic mean was considered as a video-level quality-aware feature.
8. The color gradient magnitude (CGM) map of an RGB digital image is defined as
  (6) $$CGM\left(x\right)=\sum_{c\in (R,G,B)}\sqrt{{\left(I_{x}^{c}\left(x\right)\right)}^{2}+{\left(I_{y}^{c}\left(x\right)\right)}^{2}},$$
  where the approximate directional derivatives of I(x) in the horizontal and vertical directions are denoted by $I_{x}\left(x\right)$ and $I_{y}\left(x\right)$, respectively. A video frame was characterized by the mean of its CGM, while the average of all video frames’ CGM means was considered as a quality-aware feature for a video sequence.
9. In addition to the mean of the CGM, the standard deviation of the CGM is also considered a quality-aware feature for a single video frame. As in the previous point, the average of all video frames’ standard deviation was used to characterize the whole video sequence.
10. Sharpness determines the amount of detail in an image. It is most visible in image edges, and many approaches measure it with the step response. In our model, we estimated the sharpness of a video frame by using image gradients. Namely, the gradient magnitude map (G) was calculated as
  (7) $$G=\sqrt{{\left(G_{x}*I\right)}^{2}+{\left(G_{y}*I\right)}^{2}},$$
  where $G_{x}$ and $G_{y}$ are horizontal and vertical Sobel operators, respectively. Further, I denotes an input grayscale image and * stands for the convolution operator. The sharpness of image *I* is defined as the average value of the gradient magnitude map.
11. Michelson contrast: By definition, contrast corresponds to the difference in luminance that makes an object noticeable in an image [66]. Humans tend to appreciate images with higher contrast, since they can better distinguish between differences in intensity. In our model, we incorporated two different quantizations of contrast, i.e., Michelson and root mean square (RMS) contrast. The Michelson contrast of a still image is determined as follows:
  (8) $$C_{Michelson}=\frac{I_{max}-I_{min}}{I_{max}+I_{min}},$$
  where $I_{max}$ and $I_{min}$ stand for the highest and lowest luminance, respectively. As a video perceptual feature, the average of all video frames’ Michelson contrast was taken.
12. The RMS contrast of image with size M×N corresponds to the standard deviation of intensities [67]:
  (9) $$C_{RMS}=\sqrt{\frac{1}{M\cdot N}\sum_{i=0}^{N-1}\sum_{j=0}^{M-1}{\left(I_{i,j}-\bar{I}\right)}^{2}},$$
  where $I_{i,j}$ denotes the intensity value at pixel position (i,j). Further, $\bar{I}$ stands for the arithmetic mean of all intensities. As a video perceptual feature, the average of all video frames’ RMS contrast was taken.
13. The mean of an image gives the contribution sof individual pixel intensities for the entire image. Further, the mean is inversely proportional to the haze. In our study, the average of all video frames was considered as a quality-aware feature.
14. Entropy: This can be viewed as a measure of disorder in a digital image, and at the same time, it is a statistical feature that gives information about the average information content of an image [54]. Further, entropy tends to increase in an image as the intensity of noise or degradation levels increase [68]. An 8-bit-depth grayscale image’s entropy (E) can be given as
  (10) $$E=-\sum_{n=0}^{255}p\left(n\right)\cdot \log_{2}\left(p\left(n\right)\right),$$
  where p(·) corresponds to the image’s normalized histogram count. In our model, a video sequence’s entropy corresponds to the arithmetic mean of all video frames’ entropy.
15. A perception-based image quality evaluator (PIQE) [69] is an opinion-unaware image quality estimator that does not require any training data. Further, it estimates perceptual quality only from salient image regions. First, an input image is divided into non-overlapping 16×16-sized blocks. The identification of salient blocks is carried out with the help of mean subtracted contrast normalized (MSCN) coefficients. Moreover, noise and artifact quantization are also carried out with MSCN coefficients. In our study, the average of all video frames’ PIQE metrics was considered as a quality-aware feature.
16. The naturalness image quality evaluator (NIQE) [20] is also an opinion-unaware image quality estimator that needs no training data. Namely, it quantifies image quality as the distance between the NSS features of an input image and the NSS features of a model that was obtained from pristine (distortion-free) images. The applied NSS features are modeled as multidimensional Gaussian distributions. In our study, the average of all video frames’ NIQE metrics was considered as a quality-aware feature.

### 3.2. Local Features

In our previous work, we empirically proved that the statistics of local feature descriptors are quality-aware features [14]. Further, if we apply certain human visual system (HVS)-inspired filters, dense feature vectors can be obtained. Influenced by our previous work, the following HVS-inspired image filters were applied: Bilaplacian filters, high-boost filters, and derivative filters. To be more specific, the Bilaplacian filters were motivated by the papers of Ghosh et al. [70,71], who demonstrated that the behavior of retinal ganglion cells’ extended classical receptive field can be described by a combination of three zero-mean Gaussians at three different scales, which corresponds to the Bilaplacian of the Gaussian filter. Similarly to our previous work, the following Laplacian kernels are taken into consideration:

(11) $$L_{1}=\left(\begin{array}{ccc} 0 & 1 & 0\\ 1 & -4 & 1\\ 0 & 1 & 0 \end{array}\right),\;L_{2}=\left(\begin{array}{ccc} 1 & -2 & 1\\ -2 & 4 & -2\\ 1 & -2 & 1 \end{array}\right),\;L_{3}=\left(\begin{array}{ccc} 1 & 0 & 1\\ 0 & -4 & 0\\ 1 & 0 & 1 \end{array}\right),$$

(12) $$L_{4}=\left(\begin{array}{ccc} -2 & 1 & -2\\ 1 & 4 & 1\\ -2 & 1 & -2 \end{array}\right),\;L_{5}=\left(\begin{array}{ccc} -1 & -1 & -1\\ -1 & 8 & -1\\ -1 & -1 & -1 \end{array}\right).$$

As the terminology indicates, a Bilaplacian kernel can be obtained through the convolution of two Laplacian kernels:

(13) $$L_{ij}^{2}=L_{i}*L_{j},$$

where the convolution operator is denoted by ∗. As in our previous study, $L_{11}^{2}$, $L_{22}^{2}$, $L_{33}^{2}$, $L_{44}^{2}$, $L_{55}^{2}$, $L_{13}^{2}$, and $L_{24}^{2}$ Bilaplacian kernels were applied.

High-boost filtering was motivated by the property of the HVS that it is sensitive to the high-frequency regions of a natural scene [72]. In this paper, the following kernel was used:

(14) $$H=\left(\begin{array}{ccc} -1 & -1 & -1\\ -1 & 9 & -1\\ -1 & -1 & -1 \end{array}\right).$$

Since image distortions can occur at different scales, this filter was used 4 times in succession.

Derivative filters for visual quality assessment were used first by Li et al. [73], since statistical regularities of a natural scene could be extracted by them. In our study, the following convolution of two derivative kernels was applied:

(15) $$D_{1}=\left(\begin{array}{ccc} -1 & 1 & -1\\ 1 & 0 & 1\\ -1 & 1 & -1 \end{array}\right)*\left(\begin{array}{ccc} 1 & -1 & 1\\ -1 & 0 & -1\\ 1 & -1 & 1 \end{array}\right).$$

Since image distortions can occur at various scales of an image, $D_{2}$, $D_{3}$, $D_{4}$, and $D_{5}$ in sizes of 5×5, 7×7, 11×11, and 13×13 were also applied.

Using the previously described filters, the following set of kernels can be defined:

(16) $$S=\{L_{11}^{2},L_{22}^{2},L_{33}^{2},L_{44}^{2},L_{55}^{2},L_{13}^{2},L_{24}^{2},H,H^{2},H^{3},H^{4},D_{1},D_{2},D_{3},D_{4},D_{5}\}.$$

All of the elements of the set defined by Equation (16) were applied to the *Y*, Cb, and Cr channels of an input RGB frame. The conversion from RGB to YCbCr color space could be performed by the following matrix equation [74]:

(17) $$\left(\begin{array}{c} Y\\ Cb\\ Cr \end{array}\right)=\left(\begin{array}{ccc} 0.2568 & 0.5041 & 0.0979\\ -0.1482 & -0.2910 & 0.4392\\ 0.4392 & -0.3678 & -0.0714 \end{array}\right)\left(\begin{array}{c} R\\ G\\ B \end{array}\right).$$

As a result, 3×7=21 Bilaplacian, 3×4=12 high-boost, and 3×5=15 derivative feature maps could be obtained from an input video frame. Next, FAST keypoints [15] were detected on all feature maps. Further, all keypoints were described by their 5×5 neighborhood. Each keypoint was described by a feature vector that consisted of the mean, median, standard deviation, skewness, and kurtosis of the grayscale values found in the 5×5 neighborhood. The feature vectors that characterized a feature map were obtained by concatenating the keypoints’ feature vectors. In our implementation, we set the number of keypoints to 50, since over this value, we did not experience any improvement in the performance on the KoNViD-1k [16] VQA benchmark database. As a result, a 3×7×50×5=5250 length feature vector from the Bilaplacian maps, 3×4×50×5=3000 length feature vector from the high-boost maps, and 3×5×50×5=3750 length feature vector from the derivative maps could be obtained. Similarly to the previously described global features, several statistics, i.e., mean, median, standard deviation, entropy, skewness, and kurtosis, were obtained from them to create a frame-level quality-aware feature. The arithmetic means of these statistics over time were considered as video-level quality-aware feature vectors. As a results, a vector of length 18 could be obtained for a single video sequence.

For an overview, we have provided a summary of the features introduced in our method in Table 1.

## 4. Results

In this section, our experimental results are summarized. First, descriptions of the applied datasets and the evaluation protocol are given in Section 4.1. Next, a parameter study is used to justify the design choices of the proposed method in Section 4.2. Finally, the results of a comparison with the state-of-the-art methods are given in Section 4.3.

### 4.1. Datasets and Protocol

**Figure 3:**
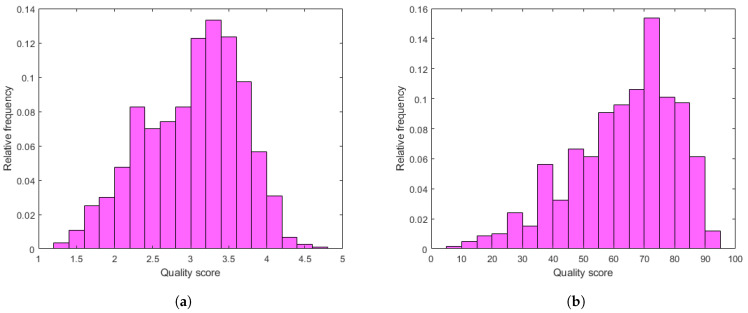
The empirical distributions of quality scores in the applied benchmark databases: (**a**) KoNViD-1k [16], (**b**) LIVE VQC [17]. The quality scores range from 1.0 to 5.0 in KoNViD-1k [16] and from 0.0 to 100.0 in LIVE VQC [17].

Experimental results and comparisons are presented on two large VQA databases that include digital videos with authentic distortions, i.e., KoNViD-1k [16] and LIVE VQC [17]. Hosu et al. [16] collected the 1200 videos found in KoNViD-1k [16] with an average length of 8 s from the YFCC100M database [75] with respect to several quality attributes, such as blur, colorfulness, contrast, spatial information, temporal information, and the numerical results of a natural image quality evaluator [20]. Quality scores for the selected videos were gathered in a crowdsourcing experiment involving 642 crowd workers from 64 countries. Further, quality scores were in the range of [1.0,5.0], where 1.0 denotes the lowest perceptual quality, while 5.0 is consistent with the highest perceptual quality. Unlike KoNViD-1k [16], LIVE VQC [17] includes 585 individual video sequences with an average length of 10 s, and the quality labels are in the range of [0.0,100.0]. The evaluation of the videos was also carried out in a crowdsourcing process with 4776 unique observers. Figure 3 depicts the empirical distributions of quality scores in the databases that were used.

As recommended in the literature, a learning-based NR-VQA algorithm was trained on approximately 80% of the videos, and it was tested on the remaining 20% [76]. The performance of an NR-VQA method is characterized by the correlation strength between the predicted and ground-truth quality scores measured on the test set. To this end, Pearson’s linear correlation coefficient (PLCC) and Spearman’s rank order correlation coefficient (SROCC) are recommended. Following the guidelines of the Video Quality Expert Group [77], scaling and nonlinearity effects between predicted and ground-truth scores were adjusted by a nonlinear transform before the calculation of the PLCC. For the nonlinear regression of scores, the following function was adopted:

(18) $$f\left(x\right)=\gamma_{1}\left(\frac{1}{2}-\frac{1}{1+\exp(\gamma_{2}\left(x-\gamma_{3}\right))}\right)+\gamma_{4}x+\gamma_{5},$$

where $\gamma_{i}$(i=1,⋯,5) are the parameters to be fitted. The equations of the applied performance metrics are as follows:

(19) $$PLCC=\frac{\sum_{i=1}^{N}\left(p_{i}-\bar{p}\right)\left(m_{i}-\bar{m}\right)}{\sqrt{\sum_{i=1}^{N}{\left(p_{i}-\bar{p}\right)}^{2}{\left(m_{i}-\bar{m}\right)}^{2}}},$$

where $m_{i}$s are raw quality scores obtained from humans and $p_{i}$s are the predictions provided by an NR-VQA algorithm. Further, $\bar{p}$ and $\bar{m}$ are mean values. The SROCC is defined as:

(20) $$SROCC=1-\frac{6\sum_{i=1}^{N}d_{i}^{2}}{N(N^{2}-1)},$$

where $d_{i}$ refers to the difference between the ranks of both measures for observation *i* and *N* is the number of observations.

To ensure the stability of the numerical results, the medians of the PLCC and SROCC are reported in this study, and they were measured over 1000 random training–testing splits. Further, the proposed method was implemented in MATLAB R2022a, and the applied computer configuration is summarized in Table 2.

### 4.2. Parameter Study

**Figure 4:**
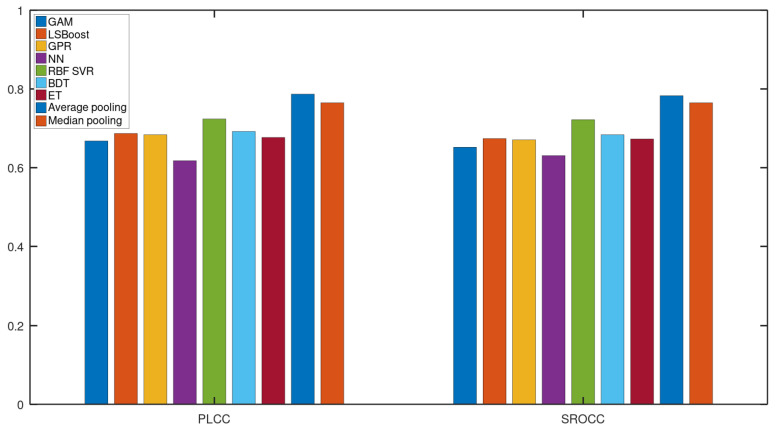
Performance comparison of different regression techniques (GAM, LSBoost, GPR, NN, RBF SVR, BDT, ET) and strategies (average pooling, median pooling) for the combination of individual regressors’ results on KoNViD-1k [16]. The median PLCC and SROCC values, which were measured over 1000 random training–testing splits, are given.

In this subsection, we justify the design choices of the proposed method. In Figure 4, a comparison of the performance of different regression techniques and strategies is depicted. The median PLCC and SROCC results were measured over 1000 random training–testing splits on KoNViD-1k [16]. From this figure, it can be seen that RBF SVR was the best single regressor, although the difference between RBF SVR and other single regressors was not too outstanding. More importantly, the mean or median pooling of the regressors’ scores resulted in a significant performance improvement.

**Figure 5:**
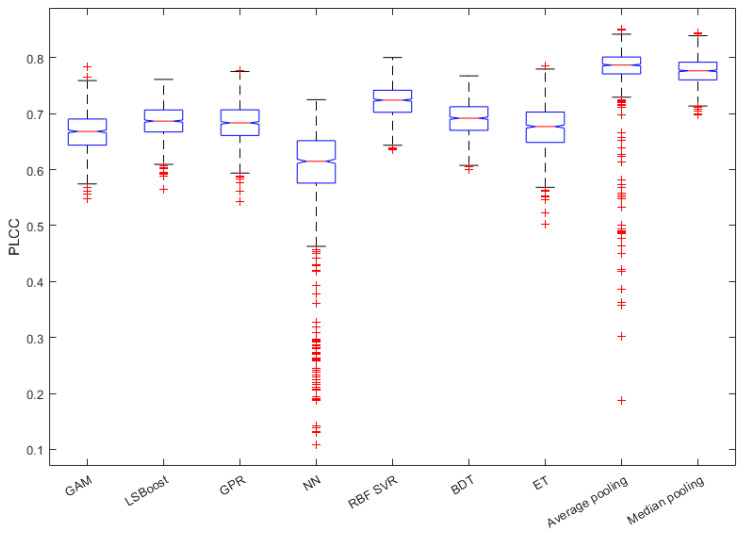
Box plots of PLCC values for different regression techniques and strategies. Measured over 1000 random training–testing splits on KoNViD-1k [16]. The bottom and top edges of each box correspond to the 25th and 75th percentiles, respectively. The whiskers continue to the most extreme data points that were not recognized as outliers, which are denoted by red ‘+’ symbols.

**Figure 6:**
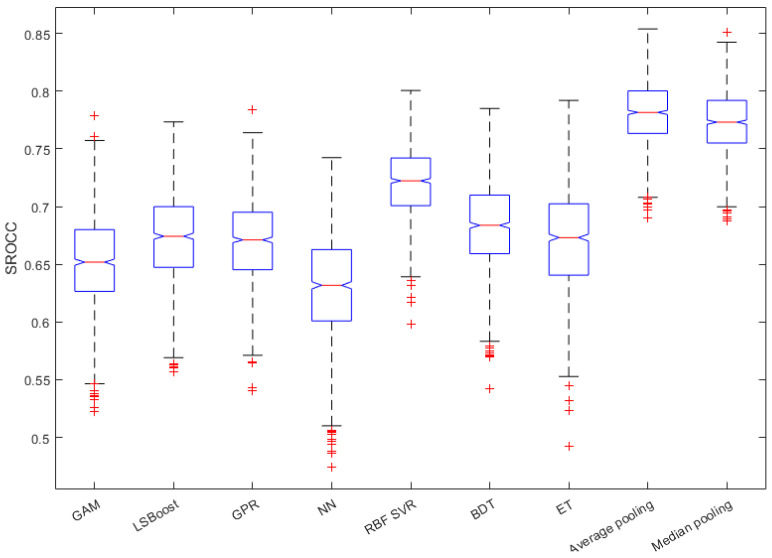
Box plots of SROCC values for different regression techniques and strategies. Measured over 1000 random training–testing splits on KoNViD-1k [16]. The bottom and top edges of each box correspond to the 25th and 75th percentiles, respectively. The whiskers continue to the most extreme data points that were not recognized as outliers, which are denoted by red ‘+’ symbols.

**Figure 7:**
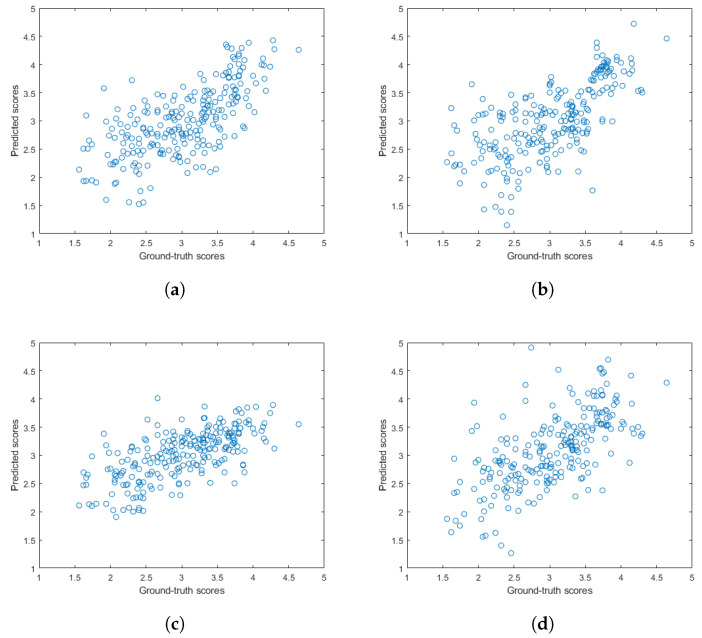

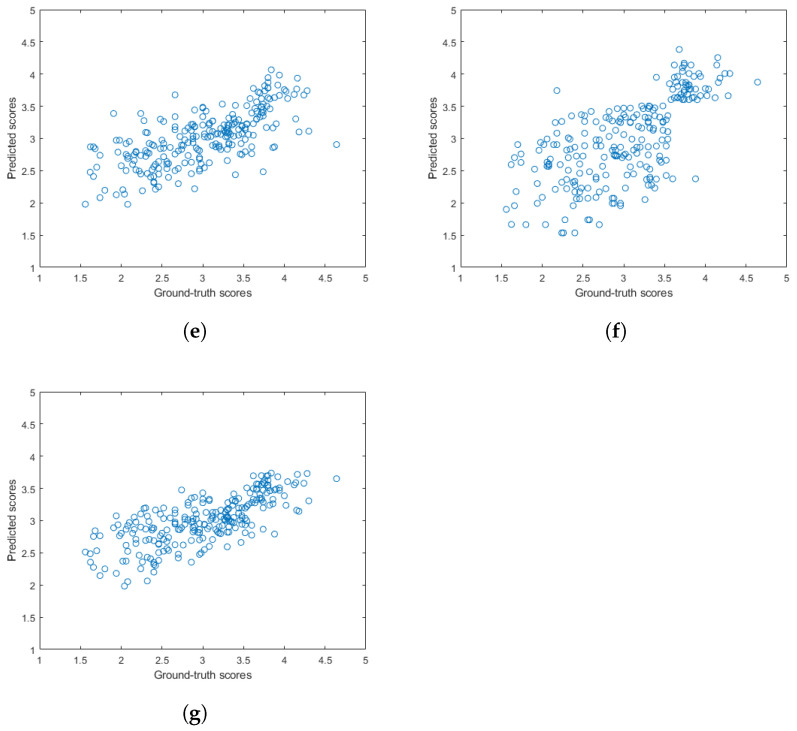
Scatterplots of the ground truth versus the predicted quality scores on a KoNViD-1k [16] test set for different regression techniques: (**a**) GAM, (**b**) LSBoost, (**c**) GPR, (**d**) NN, (**e**) RBF SVR, (**f**) BTR, and (**g**) ET.

**Figure 8:**
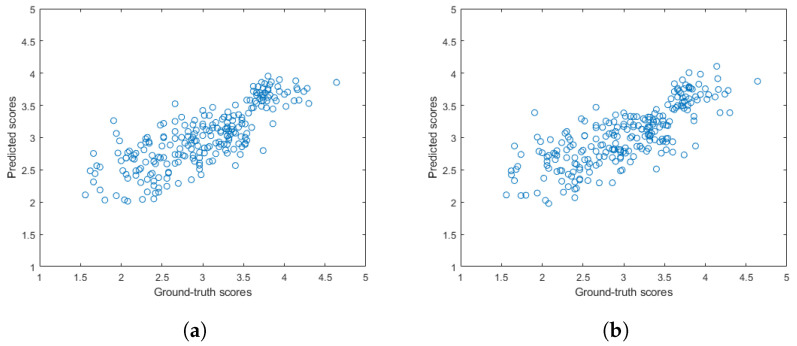
Scatterplots of the ground-truth versus the predicted quality scores on a KoNViD-1k [16] test set when using the pooling of individual regressors’ scores as a regression strategy: (**a**) average pooling, (**b**) median pooling.

Figure 5 and Figure 6 depict the PLCC and SROCC values of the different regression techniques and strategies in the form of box plots, respectively. On every box, the central mark represents the median value. Further, the bottom and top edges of the box correspond to the 25th and 75th percentiles, respectively. The whiskers continue to the most extreme data points that were not recognized as outliers, which are denoted by red ‘+’ symbols. Figure 7 and Figure 8 depict scatterplots of the ground truth versus the predicted scores on a KoNViD-1k [16] test set for each regression technique and strategy. Since the average pooling of the regressors’ scores provided the best results according to our experiments on KoNViD-1k [16], we applied this in our proposed method, which is referred to as FLG-VQA in the following, and in the comparison with other state-of-the-art methods.

**Figure 9:**
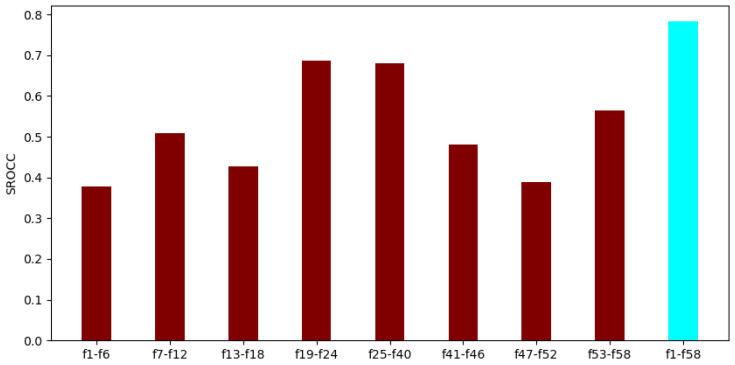
Performance comparison of the global and local features in FLG-VQA. The median SROCC values were measured on KoNViD-1k [16] over 1000 random training–testing splits. Table 1 gives information about the interpretation of the feature indices.

To demonstrate that all parts of the applied video-level feature vector in FLG-VQA are important and relevant, two additional experiments were also devised. First, the individual performance of each global and local feature was examined by using the evaluation protocol that was described in the previous subsection. The results of this experiment are summarized in Figure 9. As can be observed from these results, all global and local features were able to provide mediocre or rather strong results when considered on their own. It can be also observed that the temporal statistics of GM-LOG-BIQA [55] and the perceptual features provided the strongest individual performances, while the statistics of BRISQUE [21], SSEQ [54], and the high-boost filtered maps gave the weakest ones. The reason for this is that BRISQUE and SSEQ [54] perform better on artificial image distortions, i.e., JPEG compression noise, than on authentic distortions [14], which are found in KoNViD-1k. Further, high-boost filtering is rather sensitive to high-frequency regions in a natural scene, which may restrict its performance on extremely different authentic distortions.

**Figure 10:**
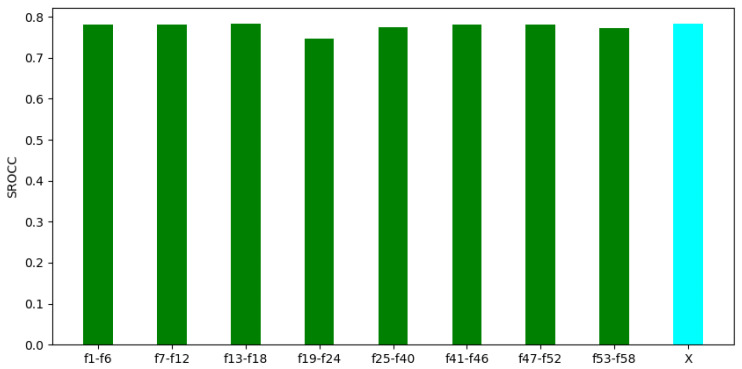
Performance of FLG-VQA in cases in which a part of the video-level feature vector was eliminated. The performance of the whole feature vector is denoted by ‘X’. The median SROCC values were measured on KoNViD-1k [16] over 1000 random training–testing splits. Table 1 gives information about the interpretation of feature indices.

In the second experiment, we made an attempt to prove that all parts of the video-level feature vector are relevant. Namely, a given part of FLG-VQA’s video-level feature vector with a length of 58 was eliminated, and then the performance of the remaining feature vector was examined. The results of the second experiment are summarized in Figure 10. From these results, it can be seen that the removal of any part of the feature vector resulted in a rather minor performance drop. Further, the removal of features that had strong individual performance did not result in a large decrease in the overall performance. Considering the experimental results in Figure 9 and Figure 10 together, it seems to be justified that all parts of the proposed video-level feature vector are important and relevant. Further, it is worth considering global and local image statistics together in VQA.

### 4.3. Comparison to the State-of-the-Art Methods

To verify the effectiveness of the proposed NR-VQA method, we compared the proposed algorithm with 10 other well-known methods, i.e., NVIE [78], V.BLIINDS [79], VIIDEO [80], 3D-MSCN [81], ST-Gabor [81], 3D-MSCN + ST-Gabor [81], FC Model [82], STFC Model [82], STS-SVR [27], STS-MLP [27], and ChipQA [83]. Specifically, the reported results of NVIE [78], V.BLIINDS [79], VIIDEO [80], 3D-MSCN [81], ST-Gabor [81], and 3D-MSCN + ST-Gabor [81] are based on our own experiments due to the availability of the original source codes of these methods. These methods were tested under exactly the same conditions as those of the proposed FLG-VQA. So, the median PLCC and SROCC values were measured after 1000 random training–testing splits, and approximately 80% of the videos were used for training, while the remaining ones were only applied in testing. The results of the other five NR-VQA methods were copied from their original publications. Further, Tu et al. [84] adapted two recently published deep-learning-based NR-IQA models, i.e., KonCept512 [85] and PaQ-2-PiQ [86], for NR-VQA. Their results, which were measured by the authors of [84], were also added to the presented comparison. Similarly to our evaluation protocol, the authors of [83,87] applied 1000 random training–testing splits and reported the median PLCC and SROCC values. Contrarily, Tu et al. [84] applied only 100 random splits, while the other papers used lower numbers of repetitions, i.e., 10 or 20. Moreover, the usual 80–20% split of the benchmark databases was used in all of the papers, since this choice is the most common and recommended for machine-learning-based methods in the literature.

The experimental results obtained on KoNViD-1k [16] and LIVE VQC [17] are summarized in Table 3 and Table 4, respectively. Further, Table 5 summarizes the results of KoNViD-1k [16] and LIVE VQC [17] in the direct and weighted averages of the performance metrics. From the presented and summarized results, it can be observed that the proposed *FLG-VQA* was able to outperform the state-of-the-art methods by a large margin. For instance, the second best, ChipQA [87], was outperformed by approximately 0.02 in terms of both PLCC and SROCC on KoNViD-1k [16]. Similarly, on LIVE VQC [17], *FLG-VQA* provided results that were 0.01 and 0.02 higher than those of ChipQA [87] in terms of the PLCC and SROCC, respectively.

## 5. Conclusions

NR-VQA, which has a high accuracy, has tremendous significance in many real-world applications. Specifically, a diverse set of local and global image features’ statistics was proposed and applied with an ensemble learning framework to obtain a perceptual quality estimator. The main consideration behind this framework was that the HVS first produces an unconscious global impression of a visual scene. Next, the HVS turns its attention to fine local details. Many quality-aware features that characterize images globally have been proposed over recent decades. We chose four of them to compile their statistics over time. Further, these statistics were boosted with several perceptual features. Moreover, local statistics were also derived with the help of three HVS-inspired filters (Bilaplacian, high-boost, and derivative filters) and the FAST keypoint detector to obtain dense frame-level feature vectors. The statistics of these dense vectors over time were considered as quality-aware features. After the fusion of the global and local statistics, an ensemble learning framework was used to map them onto perceptual quality scores. The proposed method was compared with 12 other recently published NR-VQA algorithms on the KoNViD-1k and LIVE VQC benchmark datasets. Our method’s superiority in performance was demonstrated. 

## Figures and Tables

**Table 1:** Description of features introduced in our method.

Feature Index	Description
f1–f6	Temporally pooled BRISQUE [21] statistics
f7–f12	Temporally pooled OG-IQA [53] statistics
f13–f18	Temporally pooled SSEQ [54] statistics
f19–f24	Temporally pooled GM-LOG-BIQA [55] statistics
f25–f40	Perceptual features
f41–f46	Temporally pooled Bilaplacian features’ statistics
f47–f52	Temporally pooled high-boost features’ statistics
f53–f58	Temporally pooled derivative features’ statistics

**Table 2:** Description of the computer configuration applied in our experiments.

Computer model	Z590 D
CPU	Intel(R) Core(TM) i7-11700F CPU 2.50 GHz (8 cores)
Memory	31.9 GB
GPU	Nvidia GeForce RTX 3090

**Table 3:** Comparison of *FLG-VQA* with the state-of-the-art methods on KoNViD-1k [16]. The median PLCC and SROCC values were measured over 1000 random training–testing splits. The best results are in bold, while the second-best results are underlined.

Method	PLCC	SROCC
NVIE [78]	0.404	0.333
V.BLIINDS [79]	0.661	0.694
VIIDEO [80]	0.301	0.299
3D-MSCN [81]	0.401	0.370
ST-Gabor [81]	0.639	0.628
3D-MSCN + ST-Gabor [81]	0.653	0.640
FC Model [82]	0.492	0.472
STFC Model [82]	0.639	0.606
STS-SVR [27]	0.680	0.673
STS-MLP [27]	0.407	0.420
ChipQA-0 [83]	0.697	0.694
ChipQA [87]	0.763	0.763
KonCept512 [84,85]	0.749	0.735
PaQ-2-PiQ [84,86]	0.601	0.613
FLG-VQA	**0.787**	**0.783**

**Table 4:** Comparison of *FLG-VQA* with the state-of-the-art methods on LIVE VQC [17]. The median PLCC and SROCC values were measured over 1000 random training–testing splits. The best results are in bold, while the second-best results are underlined. We indicate with “-” when the data are not available.

Method	PLCC	SROCC
NVIE [78]	0.447	0.459
V.BLIINDS [79]	0.690	0.703
VIIDEO [80]	−0.006	−0.034
3D-MSCN [81]	0.502	0.510
ST-Gabor [81]	0.591	0.599
3D-MSCN + ST-Gabor [81]	0.675	0.677
FC Model [82]	-	-
STFC Model [82]	-	-
STS-SVR [27]	-	-
STS-MLP [27]	-	-
ChipQA-0 [83]	0.669	0.697
ChipQA [87]	0.723	0.719
KonCept512 [84,85]	0.728	0.665
PaQ-2-PiQ [84,86]	0.668	0.644
FLG-VQA	**0.733**	**0.731**

**Table 5:** Comparison of *FLG-VQA* with the state-of-the-art methods using the direct and weighted averages of the PLCC and SROCC values measured on the KoNViD-1k [16] and LIVE VQC databases [17].

	Direct Average		Weighted Average	
**Method**	**PLCC**	**SROCC**	**PLCC**	**SROCC**
NVIE [78]	0.426	0.396	0.418	0.374
V.BLIINDS [79]	0.676	0.698	0.671	0.697
VIIDEO [80]	0.148	0.133	0.200	0.190
3D-MSCN [81]	0.452	0.440	0.434	0.416
ST-Gabor [81]	0.615	0.613	0.623	0.618
3D-MSCN + ST-Gabor [81]	0.664	0.659	0.660	0.652
FC Model [82]	-	-	-	-
STFC Model [82]	-	-	-	-
STS-SVR [27]	-	-	-	-
STS-MLP [27]	-	-	-	-
ChipQA-0 [83]	0.683	0.696	0.688	0.695
ChipQA [87]	0.743	0.741	0.750	0.749
KonCept512 [84,85]	0.739	0.700	0.742	0.712
PaQ-2-PiQ [84,86]	0.635	0.629	0.623	0.623
FLG-VQA	**0.760**	**0.757**	**0.769**	**0.766**

## Data Availability

The datasets used were obtained from public, open-source datasets: 1. KoNViD-1k: http://database.mmsp-kn.de/konvid-1k-database.html (accessed on 16 April 2022), 2. LIVE VQC: https://live.ece.utexas.edu/research/LIVEVQC/index.html (accessed on 16 April 2022).

## Abbreviations

The following abbreviations are used in this manuscript:

AGGD	asymmetric generalized Gaussian distribution
BDT	binary decision tree
CF	colorfulness
CGM	color gradient magnitude
CNN	convolutional neural network
E	entropy
ET	extra tree
FR-VQA	full-reference video quality assessment
GAM	generalized additive model
GPR	Gaussian process regressor
GRU	gated recurrent unit
HVS	human visual system
IQA	image quality assessment
LIVE	Laboratory for Image and Video Engineering
MOS	mean opinion score
NIQE	naturalness image quality evaluator
NMS	non-maximal suppression
NN	neural network
NR-IQA	no-reference image quality assessment
NR-VQA	no-reference video quality assessment
NSS	natural scene statistics
PIQE	perception-based image quality evaluator
PLCC	Pearson’s linear correlation coefficient
RBF	radial basis function
RMS	root mean square
RNN	recurrent neural network
RR-VQA	reduced-reference video quality assessment
SI	spatial information
SROCC	Spearman’s rank order correlation coefficient
SVR	support vector regressor
VQA	video quality assessment
VQC	video quality challenge
